# A Review of Finite Element Analysis in Spine Surgery Decision-Making

**DOI:** 10.3390/jcm15072584

**Published:** 2026-03-27

**Authors:** Elizabeth Beaulieu, Jaden Wise, Isabella Merem, Zachary Comella, Rosstin Afsahi, Joshua Roemer, Maohua Lin, Richard Sharp, Talha S. Cheema, Frank D. Vrionis

**Affiliations:** 1 College of Medicine, Florida Atlantic University, Boca Raton, FL 33431, USA; 2 Department of Biomedical Engineering, Florida Atlantic University, Boca Raton, FL 33431, USA; 3 Department of Neurosurgery, Marcus Neuroscience Institute, Boca Raton Regional Hospital, Boca Raton, FL 33486, USA

**Keywords:** FEA, finite element analysis, spine surgery, interbody fusion, adjacent segment degeneration, spinal implant design, patient-specific modeling

## Abstract

Finite element analysis is widely used to study spinal biomechanics and to compare surgical strategies under controlled loading conditions. By allowing variation in alignment, fixation, and implant design, these models provide insight into stress redistribution and motion changes that are difficult to isolate experimentally. This review examines spine surgery-focused finite element studies published between 2018 and 2024, with emphasis on interbody fusion techniques, adjacent segment mechanics, and implant-related stress behavior. Across lumbar fusion models, constructs incorporating anterior column support demonstrate lower posterior instrumentation stress than posterior-only approaches, with lateral lumbar interbody techniques showing reduced rod and screw stresses across multiple loading conditions compared with posterior lumbar interbody or posterolateral fusion constructs. In the cervical spine, comparisons of plated and zero-profile anterior cervical discectomy and fusion devices show smaller increases in adjacent-level motion and intradiscal pressure with zero-profile constructs, alongside higher localized stress at fixation interfaces. More recent studies apply finite element methods to implant optimization, alignment planning, and patient-specific modeling. Together, these findings suggest that finite element analysis is increasingly used to support surgical planning and implant design, with continued advances in validation and patient-specific simulation likely to strengthen its clinical relevance.

## 1. Introduction

The years from 2018 to 2024 represent a phase of rapid advancement in patient-specific modeling and machine learning applications. The COVID-19 pandemic in particular accelerated adoption of in silico research methodologies, including computational modeling, virtual screening, and in silico clinical trials as traditional experimental approaches faced substantial disruptions [1,2,3,4,5]. Within this evolving computational landscape, biomechanical modeling via finite element analysis (FEA) has become a cornerstone in spine surgery research, offering a powerful virtual platform to evaluate surgical strategies and implants before they are applied clinically [6,7,8,9,10,11]. By simulating spinal mechanics, FEA enables surgeons and engineers to analyze the complex mechanical behavior of the spinal column under various conditions that would be difficult or impossible to test in vivo [8]. This modeling capability is especially important given the high stakes in spine surgery: suboptimal hardware choices or fusion strategies can lead to complications such as nonunion, hardware failure, or adjacent segment degeneration [12]. Unlike cadaver studies or limited clinical trials, FEA allows systematic variation of designs and alignment parameters, helping identify biomechanical risk factors in a controlled setting [7,13]. Crucially, FEA has evolved beyond pure research; its insights are increasingly relevant for surgical decision-making. Modern spine FEA studies not only improve our understanding of normal and pathological spine mechanics but also assist in designing and selecting optimal surgical procedures and implants tailored to patient-specific anatomies [14]. In short, FEA provides a quantitative, visual, and predictive tool that can augment a neurosurgeon’s decision-making process across a range of procedures, from choosing an interbody fusion approach to planning instrumentation that minimizes future complications [14,15]. This review discusses key applications of FEA in spine surgery, compares modeling approaches, and explores current limitations and future directions, emphasizing how biomechanical simulations inform clinical choices. Key modeling characteristics of the finite element investigations included in this review are summarized in Table 1. This table focuses specifically on surgical modeling studies and does not include narrative reviews, methodological guidelines, or emerging technology perspectives cited elsewhere in the manuscript.

## 2. Methods

### 2.1. Review Design and Scope

This study was conducted as a narrative literature review examining how finite element analysis (FEA) has been applied within spine surgery research, with emphasis on construct biomechanics, implant design, adjacent segment mechanics, and patient-specific modeling applications. This work was designed and conducted as a narrative review rather than a systematic review. The intent of the search and screening process was to identify representative and methodologically informative applications of finite element modeling within spine surgery, enabling thematic synthesis of construct mechanics, modeling strategies, and surgical implications. Accordingly, study inclusion was guided by conceptual relevance and biomechanical contribution to the topics discussed, rather than by an attempt to achieve exhaustive capture of all eligible literature.

The primary review window focused on studies published between 2018 and 2024. This timeframe was selected to capture contemporary developments in spine FEA, particularly the expansion of imaging-based reconstruction workflows, improvements in computational processing capacity, and the increasing translation of modeling outputs into surgical planning investigations. Foundational studies published prior to 2018 were not excluded outright. Instead, historically significant validation studies and widely cited methodological investigations were incorporated selectively when required to contextualize modeling assumptions, benchmarking frameworks, or construct comparison methodologies referenced in modern literature.

### 2.2. Literature Search Strategy

A targeted literature search was performed using PubMed, Scopus, Web of Science, Google Scholar, and OpenEvidence, supplemented by institutional library resources and manual reference list review of relevant primary studies and prior reviews. Searches were conducted between September and December of 2025, and results were considered current through the end of the screening period.

The core Boolean search strategy combined finite element terminology with spine surgery applications. The primary search string was constructed as follows:

(“finite element” OR “finite element analysis” OR FEA) AND (spine OR spinal OR cervical OR thoracic OR lumbar) AND (surgery OR fusion OR instrumentation OR “interbody cage” OR pedicle screw OR arthroplasty OR “disc replacement” OR “adjacent segment” OR “adjacent segment disease” OR “patient-specific modeling” OR “surgical planning”).

Search filters were applied to limit results to peer-reviewed English-language publications within the defined review window. Database-specific syntax adjustments were made where necessary, but conceptual search structure remained consistent across platforms. While multiple databases were queried, the search strategy was intended to support thematic narrative synthesis rather than to function as a fully exhaustive systematic retrieval process.

### 2.3. Study Selection and Eligibility Assessment

All retrieved records were exported into reference management software for organization and duplicate removal prior to screening. Title and abstract review was performed initially to identify studies with clear surgical relevance and finite element methodology. Full-text assessment was subsequently conducted to confirm eligibility and evaluate methodological transparency.

Screening and eligibility assessment were conducted by the lead reviewing authors, each with domain familiarity in spine biomechanics and computational modeling. Titles and abstracts were reviewed collaboratively to ensure surgical relevance and methodological appropriateness, with full-text discussion used to resolve uncertainties regarding inclusion.

Studies were considered eligible if they employed finite element modeling to evaluate surgically relevant spinal constructs, instrumentation strategies, motion-preserving devices, deformity correction paradigms, or postoperative biomechanical effects. Investigations without a surgical focus, those not utilizing finite element methods, non–peer-reviewed publications, and studies lacking sufficient methodological reporting to interpret model construction or loading assumptions were excluded. Reasons for exclusion were documented during full-text review to improve transparency of the selection process.

Across the screening process, a total of 216 records were identified. Following duplicate removal and title/abstract screening, 121 studies underwent full-text review, of which 74 met final inclusion criteria and were incorporated into the qualitative synthesis.

Consistent with the narrative design of this review, eligibility decisions emphasized studies demonstrating clear surgical applicability and sufficient methodological transparency to permit biomechanical interpretation. Because inclusion was guided by thematic and clinical relevance, the potential for selection bias and incomplete literature capture remains inherent to the review structure.

### 2.4. Data Extraction and Synthesis

Data extraction was performed with a focus on variables most relevant to surgical interpretation rather than purely computational methodology. Extracted parameters included modeled spinal levels, pathological conditions, construct configurations, implant materials, loading conditions, validation strategies, and reported biomechanical outcomes such as range of motion, intradiscal pressure, facet joint forces, and implant stress distributions. Findings were organized by surgical application to allow biomechanical observations to be interpreted within operative decision-making contexts.

Given the heterogeneity of modeling strategies and reporting formats, formal meta-analysis was not pursued. Instead, trends were synthesized descriptively, with emphasis placed on reproducible biomechanical patterns observed across independent modeling frameworks. As such, the synthesis focused on directional biomechanical trends and construct-level behavior patterns across studies, rather than on completeness of study capture or pooled quantitative effect estimation.

### 2.5. Reconstruction of Quantitative Data and Figures

Several summary figures were developed to illustrate comparative biomechanical trends across the included literature. The majority of these visualizations were constructed by synthesizing numerical data reported across multiple studies, including tabulated values and stated biomechanical outcomes, and consolidating these findings into unified graphical formats to facilitate cross-study comparison. No previously published figures were reproduced.

In a limited number of instances, figures were generated using numerical data reported within a single source study when those data were presented descriptively but not already visualized in figure form. In these cases, the reported values were reformatted into original graphical displays to improve clarity and consistency across the review. These figures reflect the findings of the source study but were created independently for the purposes of this manuscript.

All visualizations are intended to illustrate relative biomechanical patterns rather than to present new primary datasets and should be interpreted within the context of the original study designs and modeling assumptions.

## 3. Key Applications of FEA in Spine Surgery

### 3.1. Interbody Fusion Techniques (TLIF, PLIF, LLIF, ACDF)

**Figure 1:**
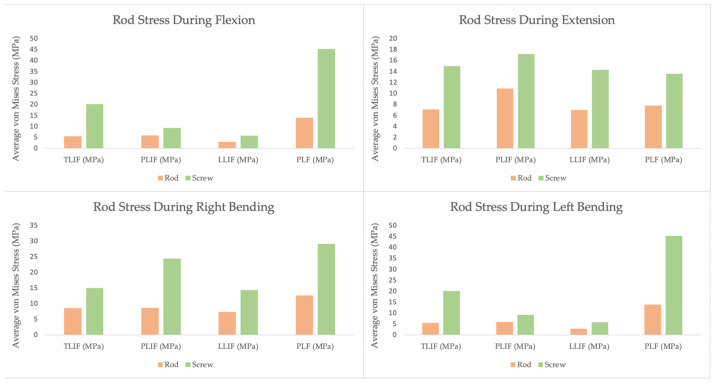
Motion-dependent rod and pedicle screw stress in lumbar fusion constructs (L1–L5), averaged across left and right sides. Data reconstructed from Shimooki et al. [1].

One major application of FEA is analyzing different interbody fusion techniques to guide surgical approach selection. Lumbar interbody fusion procedures like transforaminal (TLIF), posterior (PLIF), and lateral (LLIF) have been compared in silico to quantify spinal stability and posterior instrumentation loading, providing insight into their mechanical performance [34]. For example, one finite element study examined a multilevel L1 through L5 fusion and compared transforaminal, posterior, lateral, and posterolateral constructs by evaluating rod and pedicle screw stress under a range of physiologic loading conditions. Stress values were assessed for each motion state and averaged across the left and right sides of the construct, allowing meaningful comparison between fusion approaches [1,17]. As shown in Figure 1, PLF constructs consistently exhibited the highest posterior instrumentation stresses, whereas LLIF demonstrated lower rod and screw stresses across flexion, extension, and lateral bending. Notably, differences between fusion techniques were more pronounced during flexion and bending, while extension produced smaller inter-technique variation [18,19,35]. These findings indicate that incorporation of anterior interbody support, particularly via a lateral approach, reduces posterior instrumentation loading. Consequently, such FEA findings support the use of LLIF in longer fusion constructs to mitigate mechanical complications such as rod fracture or screw loosening.

FEA has also been used to evaluate cervical fusion strategies. In anterior cervical discectomy and fusion (ACDF), debate exists over traditional cage-and-plate constructs versus newer zero-profile integrated cages. An FEA comparing a two-level ACDF using a zero-profile device versus a cage-plus-plate found that the zero-profile implant led to smaller increases in adjacent-level range of motion and disc pressure [3]. Specifically, the zero-profile ACDF model showed the least rise in adjacent segment intradiscal pressure and endplate stress, indicating a potentially lower risk of adjacent segment disease (ASD) [3]. However, this benefit came with higher stress in the anterior cortical bone where the zero-profile anchor fits, which could increase the risk of local bone fracture. Such FEA-derived biomechanical trade-offs guide implant selection: while a zero-profile ACDF may better preserve adjacent level motion, patients with osteoporosis or poor bone quality may benefit more from a plated construct, which distributes load over a broader surface and provides greater security [3].

Patient-specific lumbar models have demonstrated that insufficient restoration of lordosis can dramatically alter postoperative biomechanics [36,37]. Abnormal sagittal alignment (e.g., residual hypo-lordosis after lumbar fusion) increases stresses and strains at adjacent intervertebral discs [13]. This finding underscores why surgeons must aim for patient-specific optimal lordosis during fusion. To aid such planning, Nikkhoo et al. developed a geometrically parametric FE model that can be rapidly morphed to different lumbar curvatures. Their simulations showed that selecting a proper cage angle to restore lumbar lordosis reduces adjacent-level stress, supporting the clinical emphasis on sagittal balance [13]. Similarly, in cervical spondylotic myelopathy, an FE model incorporating ACDF (fusion) versus alternative decompression (laminoplasty) indicated that fusion achieved spinal cord decompression but at the expense of increased strain in adjacent levels of the cord [16]. These studies illustrate how FEA compares surgical approaches (posterior vs. lateral fusion, plated vs. zero-profile ACDF, extent of lordosis correction) in silico, guiding surgeons toward techniques that optimize mechanical stability. These findings should be interpreted in the context of model assumptions, including simplified material behavior and loading conditions.

### 3.2. Adjacent Segment Degeneration

Adjacent segment degeneration (ASD) is a well-recognized long-term consequence of spinal fusion and is thought to arise, in part, from altered load transfer and compensatory motion at levels adjacent to the fused segment [38]. Finite element analyses have been widely used to evaluate these biomechanical effects by quantifying changes in facet joint forces and range of motion (ROM) at adjacent levels following fusion [20,21,39].

**Figure 2:**
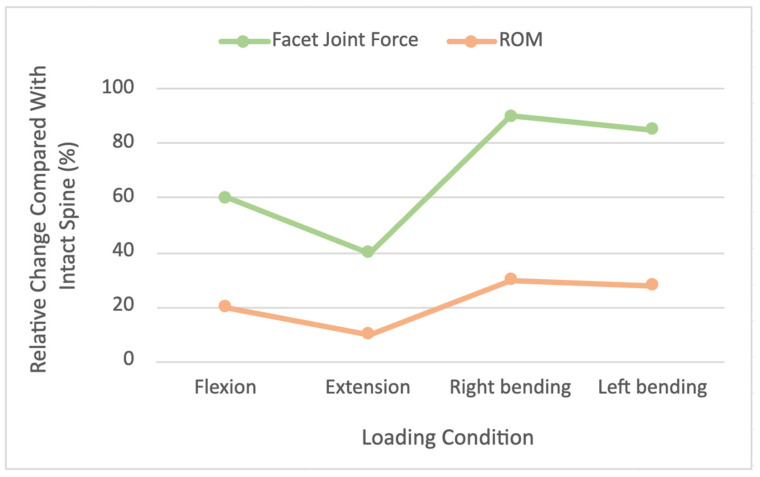
Adjacent segment facet load and range of motion changes following lumbar fusion.

As illustrated in Figure 2, finite element models consistently predict relative increases in both adjacent-level facet joint forces and range of motion following lumbar fusion across all loading conditions when compared with the intact spine. The largest relative increases are observed during lateral bending, whereas flexion and extension demonstrate more modest but persistent elevations. Similar motion-dependent increases in adjacent-level mobility following lumbar fusion have been reported in other finite element studies [40]. These motion-dependent trends highlight how fusion alters adjacent-level mechanical behavior in a manner that is not uniform across loading modes.

The use of interbody cages versus purely posterior fusions significantly influences load distribution at junctional segments. In a simulation of adult scoliosis correction involving long posterior instrumented fusion, Wang et al. demonstrated that fusion decreased motion at segments immediately adjacent to the construct while increasing stress within the adjacent facet joints [21]. As illustrated in Figure 2, these increases in facet loading were motion dependent and occurred despite modest reductions in adjacent disc pressure due to reduced segment motion [21]. This pattern suggests that while fusion stiffens treated levels, it may transfer load preferentially to posterior elements at the junctional segment, providing a biomechanical explanation for clinical observations of facet degeneration or proximal junctional pathology following fusion [4]. Notably, Wang et al. also compared different pedicle screw fixation strategies and found that the overall pattern of adjacent-level stress redistribution was preserved despite differences in construct rigidity, indicating that adjacent segment effects are driven more by fusion-induced kinematic constraints than by screw density alone [21]. Such insights can help surgeons anticipate which tissues are most susceptible to degeneration after fusion and tailor surveillance or prophylactic strategies accordingly.

Van Rijsbergen et al. developed a mechanobiological FEA framework coupling disc degeneration and bone remodeling algorithms to simulate 2 years of post-fusion changes [22]. In a patient cohort, their patient-specific models reasonably predicted which adjacent level would show disc height loss and bone density increases on follow-up imaging [22]. The direction and magnitude of bone density gains in adjacent vertebrae and degeneration grade of adjacent discs correlated with clinical observations at 12–24 months [22]. This is a striking example of how FEA can not only clarify the biomechanical basis of how ASD occurs, but also anticipate its progression in patients. Such predictive models could eventually enable personalized planning, such as identifying a patient whose L4–L5 disc would rapidly degenerate after an L5–S1 fusion, prompting discussion of prophylactic measures or a motion-preserving alternative at L4–L5.

Adjacent segment effects are amplified in long fusion constructs for deformity correction. A large-scale FEA by Nguyen et al. “virtually fused” T10–pelvis in 250 subject-specific models to study proximal junctional kyphosis (PJK) [23]. Across these virtual patients, fusion consistently decreased compressive load at T9 (the first free level above) but significantly increased shear forces at T9 [23]. In other words, after a long fusion, the adjacent segment bears more translational stress despite carrying less weight. Increased shear combined with altered motion is a known risk factor for PJK/PJF. The FEA concluded that all patients with a T10–pelvis fusion are predisposed to higher shear at T9, independent of baseline mobility. These findings suggest that prophylactic interventions, including ligament augmentation or proximal extension of the fusion, may offer benefit across a wide range of patients [23]. This large simulation provides a biomechanical rationale for the clinical observation of PJK and underscores FEA’s role in understanding and potentially preventing adjacent-level complications. These findings should be interpreted in the context of model assumptions, including simplified material behavior and loading conditions.

### 3.3. Spinal Fixation and Implant Design

FEA is extensively used to evaluate spinal fixation devices—pedicle screws, rods, plates, and interbody cages, to optimize their design and usage. By modeling bone-implant constructs, researchers can map stress distributions in both the hardware and surrounding bone, critical for predicting failure modes like screw pullout or rod fracture [24,25,26]. For instance, Sensale et al. created patient-specific lumbar vertebra models with pedicle screws to test how screw geometry (length and diameter) influences fixation stability [5]. Their FE simulations showed that increasing a screw’s diameter has a greater biomechanical impact than increasing its length: larger diameter screws significantly reduced screw deflection (by ~30%+) and lowered peak metal stress, but at the cost of higher strain in the bone around the screw [5]. This quantifies a trade-off: a thicker screw is mechanically sturdier (less likely to bend or break) but may risk bone damage or loosening in poor-quality bone. Such data helps surgeons choose appropriate screw sizes for a given patient; for example, in osteoporotic bone, an overly large screw might predispose to peri-screw bone resorption or fracture, so a moderate diameter with cement augmentation could be preferable. Notably, the same study showed that a simplified cylindrical screw model in FEA still produced accurate trend results as long as the mesh resolution was sufficiently high [5]. This is encouraging for practical, patient-specific FEA, because even with geometric simplifications, models can reliably inform implant selection [24,25,26].

Rod and instrumentation configurations have also been compared via FEA to gauge mechanical performance. The distribution of stress in spinal rods and screws during various motions indicates risk of hardware failure. As noted earlier, Shimooki et al.’s multi-level fusion model compared fusion strategies and pinpointed where the highest construct stresses occurred. In all fusion models, the peak von Mises stress in rods and screws occurred at the upper and lower ends of the instrumented span (e.g., top screws at L2 and bottom screws at L5). This matches clinical experience that screws at the ends of long constructs are prone to loosening or failure. Interestingly, adding interbody support (TLIF/PLIF/LLIF) significantly reduced rod stress compared to a pure posterior fixation (PLF), highlighting that interbody cages share load and protect the rods. Among interbody techniques, LLIF produced the lowest rod stress in flexion/extension, correlating with its larger cage footprint and better disc height restoration. These results directly inform surgical choices for long fusions: opting for a lateral approach and including anterior support can prolong implant longevity by reducing rod strain, and extra caution or reinforcement may be needed at terminal instrumented levels where stress concentrates.

FEA is equally valuable in designing novel spinal implants. Additive manufacturing has enabled complex cage designs (e.g., lattice or “truss” cages) whose performance can be evaluated in silico before clinical use. One study examined a 3D-printed interbody cage with an internal truss structure designed to promote load-sharing and bone growth [27]. By varying the thickness of the cage’s internal struts in an FE model, Kiapour et al. found that thinner struts led to higher internal strain within the cage [27]. According to mechanobiological principles, such strain can stimulate bone formation. Indeed, their subsequent animal trial confirmed that cages with thinner struts achieved faster and more robust fusion in vivo (95–100% fusion rate) compared to thick-strut, low-strain cages [27]. The FEA accurately predicted this outcome: it showed an inverse relationship between strut diameter and peak strain, and the high-strain cage design corresponded to superior fusion bone growth [27]. This underscores FEA’s role in implant innovation. By visualizing stress/strain distribution, designers can refine implant geometry to harness favorable biomechanics, such as encouraging load bearing by bone graft, while minimizing excessive stress that could lead to implant failure. In this case, FEA identified a cage design that promotes faster fusion and reduces nonunion risk while remaining within acceptable material stress limits [27].

Spinal fixation analyses also extend to special scenarios like vertebral tumors or fractures. When anterior column integrity is compromised (e.g., osteolytic tumor), surgeons must decide how best to stabilize the spine: via an anterior reconstruction (corpectomy and cage) or posterior instrumentation. FEA provides comparative data to inform such decisions. Nevzati et al. simulated a thoracolumbar spine with a destabilizing vertebral lesion and tested two stabilization strategies: a direct lateral corpectomy with cage vs. a long posterior fusion spanning the lesion [15]. The models revealed that a standalone long posterior construct without anterior support experienced higher stresses in rods and screws and transmitted more load to adjacent intact vertebrae [15]. In contrast, the anterior cage (corpectomy) construct offloaded the posterior hardware, but increased stress at the vertebrae where the cage was anchored [15]. Notably, one configuration (“Model A”) showed the lowest stress on instrumentation and neighboring levels, but higher stress in the immediately instrumented vertebral bodies [15]. The results indicate that if the vertebra can accept a cage and an anterior approach is achievable, an anterior reconstruction can better preserve overall alignment and reduce hardware burden. However, if the bone is too compromised to support a cage, a long posterior fusion may offer greater safety despite the modest increase in rod stress. The authors explicitly noted the clinical relevance: such FE data can “help with the choice of appropriate reconstruction techniques based on patient-specific characteristics” of the lesion and bone quality [15]. In summary, FEA comparisons of fixation methods, ranging from pedicle screw sizes to cage designs and construct configurations, provide essential mechanical insight that allows surgeons to tailor their technique and implant choices to maximize stability and reduce the risk of failure in each unique case. These findings should be interpreted in the context of model assumptions, including simplified material behavior and loading conditions.

Clinical Implication: FEA guides implant choices and design by revealing stress trade-offs which has been validated with in vivo studies supplemented by purely computational results. Surgeons can use FEA data to pick screw sizes and cage types that best suit a patient’s bone quality and alignment goals. Similarly, engineers rely on FEA to refine implants (like lattice cages) that achieve desired load-sharing. The result is surgical constructs optimized for mechanical durability and biological success, based on quantitative evidence rather than trial-and-error.

## 4. Evaluation of Stress Distribution and Failure Risk

**Figure 3:**
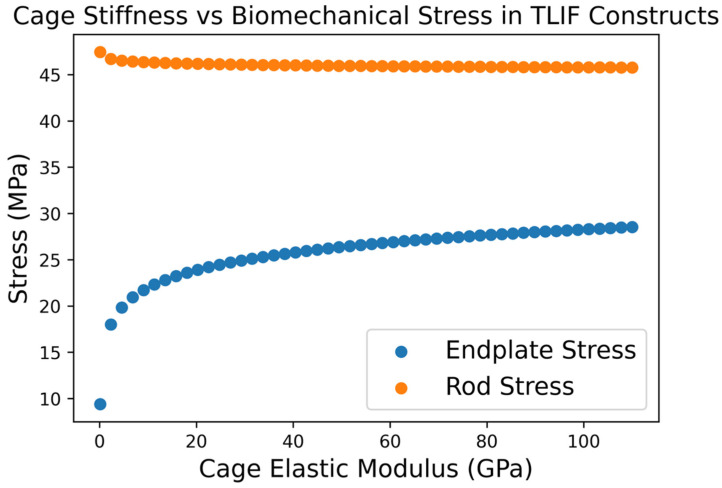
Relationship between cage elastic modulus and biomechanical stress distribution in TLIF constructs. Increasing cage stiffness is associated with higher endplate stress and reduced posterior instrumentation stress, reflecting a trade-off between subsidence risk and hardware protection. Data are reconstructed from logarithmic regression relationships derived from parametric FEA. Adapted from Lu et al. [28].

A core strength of FEA is the ability to map stress and strain distributions in spinal constructs, thereby identifying potential failure points or high-risk conditions for implants and tissues. By pinpointing where stresses concentrate, FE models can warn of likely implant failure modes (e.g., screw breakage, cage subsidence) and suggest modifications to mitigate those risks. One illustrative application is analyzing interbody cage material properties. Cage subsidence (implant sinking into the vertebral endplate) is a known complication often related to the cage’s stiffness relative to bone. Lu et al. performed a parametric FEA on a lumbar TLIF construct, varying the elastic modulus of an interbody cage from very low (0.1 GPa, akin to cancellous bone) to very high (110 GPa, titanium) [28]. They found that as cage stiffness increased, the load carried by the cage and adjacent endplates rose dramatically, whereas stress in posterior fixation and graft material decreased [28]. Specifically, peak stress in the cage and endplate was minimal when the cage was very compliant and increased nearly ten-fold with an ultra-stiff cage. Conversely, softer cages led to higher rod/screw interface stresses and more motion at the fusion site, risking hardware loosening or pseudarthrosis [28]. This relationship is illustrated in Figure 3, which demonstrates opposing trends in endplate and posterior instrumentation stress as cage stiffness increases. These simulations confirm the trade-off: a stiff cage protects posterior instrumentation but overloads the anterior column (raising subsidence risk), whereas a compliant cage reduces subsidence risk but transfers more demand to rods and screws. The study provided concrete guidance: intermediate-modulus cages strike a balance, and the authors even derived regression equations to predict the effect of any cage stiffness on key metrics [28]. For surgeons, this means material matters, i.e., an overly stiff cage in a multilevel fusion might predispose to endplate fracture or cage breakage, while an overly flexible cage might necessitate more robust posterior fixation to prevent failure. Because of FEA, such cause-and-effect relationships between implant material and failure risk are quantified, enabling evidence-based choices of cage material (PEEK vs. titanium vs. novel composites) depending on the scenario [28].

FEA also helps identify subtle “stress risers” that could lead to implant fatigue or loosening over time. In evaluating posterior fixation, models often show that stress is not evenly distributed: certain screws or rod segments consistently bear more load. For example, as noted earlier, multi-level fusion FEA showed that the greatest rod stress appears at the junctions where the fused construct meets unfused segments, and the highest pedicle screw stress occurs in the most superior and most inferior screws. With this information, a surgeon may choose to add prophylactic features such as transition rods or hooks at the top of a long fusion to better distribute stress. Alternatively, a surgeon could choose a rod material with higher fatigue strength if a high-stress concentration is unavoidable. Similarly, cervical spine models comparing disc arthroplasty with fusion have quantified facet joint forces and shown that a mobile artificial disc preserves more natural load sharing than fusion [8]. This has direct implications for failure risk, since excessive facet loading can lead to hypertrophy and pain, and an implant that generates abnormal facet contact forces may drive facet degeneration, thereby undermining the goal of motion preservation. By comparing designs with FEA, engineers can favor an artificial disc that minimizes facet stress, reducing the likelihood of adjacent facet pathology.

Another contribution of FEA is predicting failure risk for the spine itself. Case-specific models with patient-specific bone density distributions have been used to predict fracture risk under loads. Groenen et al. developed nonlinear FE models of spinal segments with metastatic lesions and compared predicted failure load to experimental tests [29]. The FE models correctly identified which vertebra would fracture in 10 of 11 tested specimens, indicating good predictive power in localizing failure [29]. However, the absolute load to failure predicted by the models did not correlate strongly with actual failure loads (R^2^ ~0.22) [29]. This partial success highlights both the promise and current challenge of FEA in failure risk assessment. These models can reveal the segment most likely to fail, such as a lytic L2, but defining the true safety margin of that segment is far more challenging. Still, the ability to flag the weakest vertebra is clinically valuable. In metastatic spine cases, such an FE analysis could influence the surgical plan. For example, reinforcing a vertebra that the model predicts is likely to collapse under physiological loads. Moreover, these studies spur model improvements. Groenen et al. noted that including the disc and posterior elements, rather than an isolated vertebra, was key to correctly predicting fracture location. They also reported that using different bone density–strength relationships produced similar failure predictions [29]. Insights like this steadily improve FEA’s reliability as a tool for assessing structural failure risk in spine surgery patients [29].

In summary, through detailed visualization of stress distributions and evaluation of alternative scenarios, FEA helps spine specialists recognize critical warning signs, such as an implant approaching its stress limits, a bone–implant interface becoming unstable, or an unfused segment taking on excessive load after surgery. These findings translate into risk mitigation strategies—choosing materials of appropriate stiffness, augmenting or redesigning implants to eliminate stress concentrators, and planning constructs that avoid subjecting any one component to excessive load. The ultimate goal is fewer mechanical failures postoperatively, guided by the foresight that computational modeling provides.

Clinical Implication: FEA-driven stress analysis allows surgeons and device makers to foresee failure risks and adjust accordingly. For example, understanding that a titanium cage can cause subsidence may lead a surgeon to opt for a PEEK cage or supplementary support. Recognizing high stress on a particular screw might prompt using a larger screw or adding cement. In essence, FEA “stress-testing” of a plan can inform preemptive measures that improve implant longevity and patient outcomes. These implications are, however, purely computational.

## 5. Comparative Analysis of FEA Models and Clinical Utility

### 5.1. Model Types and Accuracy

Not all FEA models are created equal: differences in modeling assumptions and complexity influence how well they predict surgical outcomes. A recent theme is balancing simplicity with physiological realism. Traditional spinal FE models often apply simple boundary conditions (e.g., pure moments with a generic compressive preload) and neglect active muscle forces, mainly for convenience [7]. While these passive models yield useful qualitative insights, studies show they may misestimate certain forces. Abbasi-Ghiri et al. highlighted this by comparing a “traditional” passive lumbar FE model (constant generic load) against a “novel” FE model informed by a musculoskeletal simulation, which provided level-specific, posture-specific muscle forces [7]. The novel force, displacement controlled FE model, driven by realistic vertebral rotations and tailored follower loads, predicted adjacent-segment disc compression, intradiscal pressure, and ligament forces much more accurately relative to a detailed musculoskeletal model (treated as gold-standard) [7]. In contrast, the traditional FE model (with generic static load) showed significant errors in those metrics, especially failing to capture shear forces and facet loads accurately [7]. The take-home message is that FEA driven by more biofidelic loading conditions (including muscle effects or patient-specific kinematics) substantially improves predictive fidelity. As a result, the authors caution that clinical recommendations based solely on simplistic FE models should be interpreted with caution [7]. This kind of comparison is prompting a shift toward more sophisticated “hybrid” modeling (combining musculoskeletal and FE methods) to enhance clinical reliability of FEA predictions.

Another comparative aspect is personalized FE models versus generic ones. Historically, many spine FE studies used one “average” geometry (often from a single cadaver or scan) to draw general conclusions. For instance, soft-tissue components are incorporated using generalized anatomical assumptions, particularly when CT imaging serves as the sole data source [41]. However, patient anatomy varies widely, and a model accurate for one morphology might not apply to another. Model personalization thus becomes inherently limited, relying on “universal” human anatomy to fill in missing information. While MRI improves soft-tissue visualization, it does not directly yield patient-specific mechanical properties either. Although landmark-based mesh deformation methods demonstrate that generalized musculoskeletal templates can approximate subject-specific geometry with millimeter-scale accuracy, these approaches remain constrained by manual landmark selection, smoothing assumptions, and a focus on geometric rather than physiologic personalization [42]. The need for customization is now recognized: “most FEA studies used only one unique spine model,” which is a limitation for clinical translation [13,14]. Recent efforts like Nikkhoo et al.’s parametric modeling system aim to overcome this by automatically morphing a baseline FE mesh to match each patient’s X-ray geometry [13]. When applied to 10 patients’ lumbar spines, their FE-predicted motions and disc pressures post-fusion varied according to each patient’s lordosis, and the model results aligned well with each individual’s imaging data [13]. This suggests personalization captures important biomechanical differences that a one-size-fits-all model would miss. Importantly, the authors demonstrated their streamlined model (based only on upright lateral X-rays) was efficient and reproducible in a clinical workflow—two clinicians could independently generate model measurements with good reliability [13]. Such developments indicate that going forward, model evaluations will focus on clinical deployability: models must be fast, user-friendly, and adaptable to individual patients to truly impact surgical planning. In short, the trend is toward comprehensive, customizable models (including patient-specific anatomy and more physiologic loading) instead of static generic simulations, because the former demonstrably improve prediction accuracy and clinical relevance [7,13,14,30,41,42,43,44,45,46,47,48,49,50,51,52].

Comparisons have also been drawn between FEA predictions and real-world outcomes to test model effectiveness. We saw examples like the lattice cage study and the adjacent degeneration prediction study where FEA outcomes were directly validated by in vivo results [22,27]. These are essentially case-by-case checks: does the model predict what actually happens? In the cage example, FEA correctly predicted the superior fusion success of the thinner-strut cage, boosting confidence in that model’s biomechanical criteria for fusion. In the adjacent degeneration example, the model’s reasonable match with 2-year follow-ups showed that coupled disc/bone remodeling algorithms in FEA can mirror clinical degeneration trajectories. However, other comparisons highlight where models still fall short—for instance, Groenen’s work where FE predicted fracture location well but not magnitude of failure load [29]. Such mixed results underscore that continued refinement and calibration of FE models are necessary. A 2023 study by George et al. tackled this by calibrating and validating a lumbar spine FE model against multiple experimental datasets [30]. They adjusted their model until it simultaneously matched flexion-extension ROM corridors from six different cadaver studies and reproduced measured disc pressures and facet contact forces within physiological ranges [30]. The outcome was a highly validated model serving as a reliable baseline for testing fusion constructs [30]. This rigorous validation is a benchmark for clinical-grade modeling, only when an FE model is proven accurate in multiple metrics can we fully trust its predictions in new scenarios. Thus, comparative analysis in the FEA field is as much about comparing predictions to experimental/clinical reality as it is about comparing different models to each other. Both drive improvements in modeling techniques and boost the credibility of FEA as a decision-support tool [22,27,29,30].

Across the included literature, validation strategies varied substantially and can be broadly grouped into three categories: experiment validation against cadaveric mechanical testing, in vivo or longitudinal clinical comparison, and benchmarking against previously validated computational models. Cadaveric validation remains the most direct biomechanical confirmation approach, with studies comparing predicted range of motion, disc pressure, or failure location against laboratory loading experiments [29,30]. These investigations provide higher mechanical fidelity but are limited by specimen variability and controlled testing environments. In contrast, in vivo or longitudinal validation strategies, such as mechanobiological modeling of adjacent segment degeneration or implant performance studies, evaluate predictive correspondence with clinical imaging or fusion outcomes over time [22,27]. While these approaches offer stronger translational relevance, they often involve smaller cohorts and indirect biomechanical endpoints. A third category involves internal benchmarking against previously validated FE frameworks or multi-study ROM corridors to ensure consistency with established biomechanical ranges [30]. Although this approach improves computational robustness, it does not substitute for experimental or clinical validation. Taken altogether, the heterogeneity of validation approaches highlights both the maturation of the field and the ongoing need for standardized reporting and multi-level validation frameworks that integrate experimental, computational, and clinical outcome data.

### 5.2. Traditional Finite Element Analysis Versus AI-Enhanced FEA

To compare the relative strengths and limitations of conventional finite element analysis (FEA) with artificial intelligence-enhanced FEA (AI-FEA), we assessed six domains that reflect both biomechanical and translational relevance: bone density integration, comorbidity inclusion, dynamic loading simulation, computational speed, predictive adaptability, and patient specificity. This domain-based comparison is intended as an interpretive, literature-informed framework rather than a formally validated quantitate scoring system. The six domains were selected to reflect recurring themes in contemporary publications addressing translational and biomechanical limitations of conventional FEA. The comparative discussion is therefore exploratory and conceptual, designed to highlight emerging methodological directions rather than to provide a reproducible quantitative ranking of modeling approaches.

Traditional FEA incorporates bone density information using CT-derived Hounsfield units, which are converted into elastic material properties through predefined mapping relationships. However, these models assume isotropic, linear elastic bone behavior, involve calibration uncertainty, and often fail to capture patient-specific or pathological variations in tissue mineralization [8,43].

Furthermore, conventional FEA focuses almost exclusively on mechanical behavior and does not incorporate physiological risk factors that influence bone quality or healing, such as metabolic health, medications, and age-related changes in tissue quality [44]. Furthermore, although traditional FEA can simulate axial, bending, and torsional loads, its capacity for dynamic loading simulation remains limited. These loading conditions are typically static and user-defined, lacking the ability to adapt to evolving physiologic patterns [8,45]. The boundary conditions applied in virtual mechanical testing are often simplified representations of physiological loading and may not fully reproduce the complex, time-dependent mechanical environment associated with real-world activities, such as walking or falling [46]. Consistent with this limitation, traditional FEA demonstrates limited predictive adaptability and patient specificity, as models typically rely on fixed assumptions of ideal axial, bending, and torsional loading scenarios, single-anatomy representations, and population-averaged tissue properties [46,47]. Additionally, traditional FEA is computationally intensive, requiring substantial time for high-fidelity meshing, material-property assignment, and iterative solver convergence to ensure numerical accuracy [48]. This often results in delays of hours to days for complex anatomical structures, rendering traditional FEA impractical for real-life clinical decision making [49]. In fact, Mesh refinement studies demonstrate that stress and strain distributions may not converge at element sizes as small as 1–2 mm, requiring individual convergence analyses that add computational burden [50].

In contrast, AI-FEA frameworks integrate machine learning algorithms, surrogate solvers, and multimodal clinical datasets to address many limitations of traditional FEA. For instance, AI-driven multimodal models can incorporate demographic, metabolic, and clinical risk variables into predictive frameworks, extending beyond purely mechanical analysis and incorporating patient risk factors into outcome predictions [51]. AI-FEA adaptive learning and physics-informed neural networks also allow for a more realistic, time-dependent modeling of physiologic and traumatic conditions [51].

Furthermore, AI-driven surrogate solvers and reduced-order models can reproduce FEA outputs in real time, overcoming the hour-long computation time required for traditional finite element simulations [49,52]. Continuous model retraining allows these systems to evolve with expanding datasets and support individualized digital twin simulations that reflect patient-specific anatomy and material heterogeneity [51,53]. Finally, machine learning and physics-informed neural network pipelines reduce reliance on manual segmentation, mesh refinement, and mapping of CT Hounsfield units to elastic material properties [51]. For instance, automated segmentation can differentiate trabecular from cortical bone with manual and automatic segmentations agreeing within approximately one voxel (0.21–0.99 mm) [54].

Overall, contemporary literature suggests that AI-enhanced frameworks may offer advantages in computational efficiency, adaptability, and personalization relative to conventional FEA approaches, particularly in scenarios requiring rapid or large-scale simulation. However, these advantages remain dependent on dataset quality, validation rigor, and appropriate integration with established biomechanical principles. It is important to emphasize that AI-FEA methodologies remain heterogeneous and are at varying stages of validation. Many reported improvements are demonstrated within controlled research environments and may not yet generalize across diverse clinical populations. Accordingly, AI-enhanced approaches should currently be viewed as complementary to, rather than replacements for, rigorously validated conventional finite element workflows.

Clinical Implication: Understanding the accuracy limits of different FEA models prevents over-reliance on simulations. Surgeons and researchers should favor FE models validated against physical data and reflective of patient-specific conditions. In practice, this means one should give more weight to, say, a personalized, muscle-driven FEA prediction than to a generic static model’s output. As models become faster and more evidence-backed, their clinical utility in planning surgeries will grow, bridging the gap between engineering and operative care. These results are purely computational and should be considered with appropriate caution.

### 5.3. Advantages and Limitations in Practice

FEA offers several clear advantages for spine surgery planning. First, it enables virtual trials of surgical options without risk to patients [55,56,57,58]. Complex questions, such as whether a 2-level fusion will overload a third level, or how much instrumentation is needed for a tumor case, can be evaluated on the computer, reducing reliance on subjective judgment [15]. This is especially valuable for novel devices or techniques where clinical experience is limited. FEA makes it possible to rigorously test designs like cages and arthroplasty implants under physiological loading long before clinical use [14]. Second, FEA can provide quantitative insights that are hard to obtain otherwise. It can output internal stress, strain, and pressure values at virtually any point in the spine—data that even the most sophisticated imaging or intraoperative monitoring cannot directly provide. These insights help identify why a particular failure occurred or might occur, such as pinpointing a stress concentration to explain a screw failure. In one review, Wang and Wu noted that FEA has been critical in “demonstrating cage characteristics after implanting” and in lowering the learning curve for surgeons by visualizing how new cages behave biomechanically [14]. This visual, quantitative feedback can guide surgeons in selecting “the best interbody cage for patients” based on objective criteria rather than solely on anecdotal experience [14]. Third, modern FEA is increasingly patient-specific, meaning it can cater to individual anatomies and conditions. As personalized medicine rises, FEA is keeping pace. Patient CT or MRI data can be converted to FE models that reflect that individual’s bone quality, alignment, and pathology. This suggests a future where a surgeon could simulate a proposed surgery on a patient’s digital twin to predict that specific patient’s outcome, much like aerodynamic simulations are custom-run for each new aircraft design [14,15,55,56,57,58].

Despite these advantages, several limitations temper the routine clinical use of FEA today, as summarized in Table 2. One major limitation is model complexity and expertise. Building and interpreting an FE model traditionally requires specialized engineering knowledge, making it impractical in a busy clinical workflow. The “learning cost” for surgeons, having to master software or rely on engineers, has been cited as a barrier [14]. If the modeling process is too time-consuming, or the results are too difficult to interpret, most clinicians will not find it practical for daily decision-making [13]. However, initiatives to simplify the interface, such as template models that only need a few measurements from radiographs, are underway to reduce this barrier [13]. Another limitation is the inherent assumptions and simplifications in FE models. To keep computations tractable, models often assume linear elasticity for tissues, omit smaller muscles, or use average material properties [14]. These simplifications can impact accuracy. For instance, assuming bones are perfectly homogeneous and linear might misjudge failure risk in osteoporotic spines, and ignoring muscle forces might overestimate spinal motion under load [7]. Researchers are aware of these issues: one analysis tallied that neglecting musculature and assuming linear materials were the top two limitations mentioned in recent FEA papers [14]. As computational power grows, newer models are incorporating more non-linear and biologically realistic features to address these gaps, although this progress continues to be limited by the trade-off between model detail and solve time.

Validation remains a critical challenge as well. An FE model is only as good as its correlation with reality. Although many studies validate portions of the model by comparing the intact spine’s range of motion with cadaver data, far fewer verify predictions after surgery or over longer time spans [20]. As Azadi and Arjmand noted, a novel contribution of their work was validating the fused-spine model against cadaveric fusion experiments, not just the intact spine [20]. They achieved strong agreement in adjacent level motions, but disc pressures were more difficult to verify, as the predicted pressures at adjacent levels fell outside the narrow in vitro range even though the overall trends aligned [20]. This highlights that certain outputs, such as absolute disc pressure, are highly sensitive to modeling assumptions and measurement uncertainty. It highlights the need for higher quality experimental data, including more comprehensive cadaver studies or even in vivo measurements using instrumented implants, to help calibrate FE models. Without robust validation, surgeons may be skeptical of model predictions, limiting clinical adoption. After all, if a model’s prediction could be off by a large margin, one cannot confidently base a surgical plan on it. Therefore, improving validation methods through cadaver labs, animal models, or retrospective clinical data comparisons is an essential step toward building trust in FEA tools for surgeons [20].

In summary, FEA in spine surgery offers a powerful extension of the surgeon’s decision-making arsenal by revealing biomechanical information invisible to the naked eye and by allowing virtual experimentation. Its advantages in pre-testing implants, customizing to patient anatomy, and quantifying risk factors are clear from the literature and have led to significant biomechanical discoveries. However, to fully integrate FEA into clinical practice, the community must continue to address its limitations: making models faster and easier to use, including more physiological detail for accuracy, and rigorously validating predictions. As these challenges are met, the gap between engineering bench and bedside will narrow, and FEA’s clinical impact will increasingly be realized.

Clinical Implication: Surgeons should view FEA as a complementary tool—one that provides valuable insights but must be interpreted in context of its assumptions. When high-fidelity models are available, they offer a predictive “crystal ball,” helping choose between implants or plan alignment to avoid complications. Conversely, awareness of FEA’s limitations reminds us that these models are aids, not oracles; their predictions should ideally be corroborated by clinical data. The future lies in making FEA both credible and convenient for everyday surgical planning.

## 6. Challenges and Future Directions

### 6.1. Current Challenges in FEA Modeling for Spine Surgery

While the capabilities of spine FEA have expanded, several challenges continue to limit its routine clinical implementation. One persistent issue is achieving the right balance between model detail and computational efficiency. High-fidelity models that include non-linear material properties, fluid nucleus behavior, muscle forces, and subject-specific geometry can be computationally heavy and complex to set up [14]. Consequently, current models still use simplifying assumptions like linear elasticity and omit softer tissues (e.g., spinal cord, detailed facet cartilage) to make the simulations solvable in a reasonable time [14]. However, these simplifications, as noted, can sacrifice accuracy in certain scenarios. Ongoing improvements in computational efficiency are expected to mitigate this limitation, but careful interpretation of simplified models remains necessary.

Another challenge lies in model generalizability and variability. Historically, many studies have relied on single representative anatomy; this limits applicability of findings across heterogeneous patient populations [14]. Interindividual variability in anatomy, bone quality, and ligament properties can substantially influence biomechanical outcomes. To address this, population-based and statistical shape modeling approaches have emerged, but they require large imaging datasets, automation, and significant computational resources. While feasible, such approaches are not yet widely accessible in clinical environments.

Validation and integration with clinical data remain crucial challenges as well. FEA predictions must be continually compared against cadaveric experiments, in vivo measurements, or longitudinal clinical outcomes. Unfortunately, it is challenging to get such data: internal spinal loads are rarely measured in patients and long-term outcomes such as degeneration or implant failure require extended follow-up. Although creative validation strategies such as benchmarking against multiple experimental datasets [43] have shown promise, large scale prospective validation remains limited [30]. Yet, there are aspects like long-term degeneration or patient-reported outcomes that are hard to validate without clinical studies. A challenge moving forward is conducting prospective studies where patients’ surgical plans are simulated beforehand, predictions (e.g., risk of adjacent degeneration or hardware failure) are recorded, and then outcomes are tracked to see if the model was right. Until such data are more widely available, FEA predictions should be interpreted cautiously and as adjunctive rather than definitive evidence.

Reporting of spinal finite element models is governed by the FDA’s guidance for reporting computational modeling in medical device submissions. This dictates that a comprehensive report must include clear documentation of geometric acquisition, material properties, and specific boundary conditions. This is to ensure independent reproducibility [31].

Validation standards compare computational results against standardized physical testing protocols, primarily those established by ASTM International. For instance, the ASTM F1717 standard [59] provides a vertebrectomy model utilizing polyethylene blocks to simulate a worst-case mechanical environment for testing screw and rod constructs under static and fatigue loading. By replicating these standardized setups within a virtual environment, finite element models can be rigorously validated against known mechanical benchmarks, ensuring that the simulated behavior of spinal hardware correlates with established performance data before advancing to complex patient-specific simulations [60].

Finally, a pragmatic challenge is interdisciplinary communication. Many surgeons lack formal training in computational biomechanics. Improving visualization, standardizing clinically meaningful metrics, and developing intuitive decision support interfaces are essential to ensure biomechanical insights are accessible and actionable in surgical planning [14,61]. It’s as much a socio-technical challenge as a scientific one. In sum, current challenges include refining model fidelity, capturing patient variability, rigorously validating predictions, and packaging the technology for clinical use. Overcoming these hurdles will be key to FEA becoming a standard part of spine surgery planning.

### 6.2. Advancements and Future Directions

The future of FEA in spine surgery is geared toward more personalized, faster, and smarter modeling that can seamlessly integrate into clinical workflows [31]. One major direction is the development of patient-specific modeling pipelines. Instead of an engineer manually building a model over several days, future solutions may allow a surgeon to input a patient’s imaging data and receive an automated FE model analysis within hours or even minutes. The customizable lumbar model by Nikkhoo et al. is a step in this direction, using just X-ray inputs to update a baseline model [13]. Likewise, George et al.’s hexahedral mesh model emphasizes a morphological approach that can be quickly adjusted to different anatomies without losing accuracy [30]. As these methods mature, we can envision software integrated with hospital PACS systems where, for example, a surgeon planning a fusion can request a “FEA report.” That report might detail predicted ROM loss, adjacent level stress changes, and implant load safety factors for various surgical options (one level vs. two level fusion, different cage sizes, etc.), all based on that patient’s data. Such patient-specific decision support would directly embody the oft-stated goal of FEA research: to assist in determining the optimal surgical plan for each patient [14].

Another exciting advancement is the incorporation of machine learning (ML) and AI alongside FEA. ML algorithms can be trained on large numbers of FE simulations to identify patterns or even serve as fast surrogates for FEA. For instance, instead of running a full nonlinear FE simulation for every possible rod diameter in a scoliosis correction, a trained ML model could instantly predict stresses based on prior simulations. Teng Lu’s study effectively did this with a regression model, fitting a logarithmic equation to FEA results so that surgeons could plug in any cage stiffness value and get an immediate prediction of fusion biomechanics [28]. This in silico test was assessed across 23 elastic modulus values ranging from 0.1 GPas to 110 GPa at 5 Gpa intervals. This is a simple form of AI integration, the FEA generated the data, and then a regression (a basic AI model) provided a formula for instant use. Machine learning integration with FEA has also been seen to accelerate spinal implant optimization. These hybrid models can create surrogate models which can predict the biomechanical responses of the spine to varying implant types in minutes rather than hours [32]. Phellan et al. reviewed 41 publications and found that ML algorithms trained on FEM data could accelerate biomechanical simulations to real-time (milliseconds) while maintaining high accuracy, frequently measured by low mean squared error and high correlation coefficients [49]. Other studies suggest that integration can reduce implant development time by over 97% with an error margin of less than 5% [62,63]. These studies were conducted using a high-resolution CT scan of a single adult subject (L1–L5) from a public dataset to ensure analytical consistency. Simulations were performed under quasi-static physiological loading scenarios, including flexion, extension, axial rotation, and lateral bending, while the lower endplate of L5 was rigidly constrained.

Ultimately, this enables patient-specific modeling where automated segmentation and ML-accelerated simulations can generate personalized biomechanical assessments for preoperative planning [33,62,64]. This approach also facilitates multi-objective optimization; it simultaneously evaluates fracture risk, fusion rates, and postoperative stability across different bone conditions [32].

Additionally, we can expect even more sophisticated uses of AI, such as neural networks that take patient images and output likely regions of high stress or risk post-surgery without running a full FE solve [63]. Such AI models would be “trained” on many simulated scenarios. Neural networks trained on patient images and FEA datasets can predict high stress regions in seconds rather than hours [65,66]. They serve as surrogate models that learn high complex biomechanical relationships. One study by Cai et al., tested these models and they achieved mean absolute errors below 0.06 MPa for stress predictions and reduced the processing time from 90–120 min to approximately 2–3 min per subject. These models operate through several integrated steps. Convolutional neural networks are trained on large data sets which combine patient-specific CT imaging with corresponding FEA-computed stress distributions [66]. The models then encode vertebral geometry and decode stress patterns while enhances physics-informed neural networks (EPINN-GF) integrate both global and structural characteristics of the spine [67,68]. Zhang et al., demonstrated that the EPINN-GF method Theis superior to traditional collection-based methods which produced mean squared errors of 227.59 MPA and 398.41 MPa respectively. Furthermore, the combination of FEA with data-driven models enables real-time surgical planning through an automated end-to-end pipeline. Once trained, these models would be able to process new patient CT scans. Through automated segmentation and instant biomechanical prediction, these models eliminate the need for time-intensive mesh generation and iterative solving [62,67]. For surgical decision-making, this integration allows surgeons to rapidly evaluate multiple implant configurations, materials, and placement during preoperative planning. This could be especially valuable in complex cases such as adult spinal deformity; ML algorithms could establish intraoperative risk levels and predict post operative functional outcomes based on patient profiles [69,70].

Advances in model validation and multimodal data integration are also anticipated. In the future, we might see smart spinal implants that collect data on loads (there have been prototypes of instrumented rods measuring strain in vivo). These smart spinal implants are embedded with sensors which create closed-loop refinement systems by continuously collecting in vivo load data during healing and daily activities; this data is then used to update FEA models to improve their predictive accuracy over time [71,72]. The technical foundation for smart implants already exists, piezoelectric transducers can harvest energy from spinal micro-motion to power self-contained sensor-data-logger systems. These systems then record the mechanical use of fixation devices throughout the fusion process [70]. Polymetric flexible sensors and microelectromechanical systems (MEMS) show favorable attributes for in vivo load-sensing; however, extensive testing in spinal applications remain ongoing [72]. Moreover, imaging techniques like dynamic radiographs or MRI can provide patient-specific motion and tissue condition information that can initialize or update an FE model more accurately. Quantitative fluoroscopy (QF) captures vertebral displacements and rotations during functional movements like flexion and extension which can be directly applied as boundary conditions in subject-specific models [73]. This, when combined with MRI for tissue characterization, can predict stress distributions that correlate with disc orientation and wedging patterns. Du et al., explored the use of dual fluoroscopic imaging systems (DFIS). DFIS enables even more sophisticated analysis by tracking three-dimensional kinematics across seven different motions. This has revealed regional variations in intervertebral disc mechanical responses which differ substantially from generalized loading assumptions [74]. MRI has also proven a useful tool as it contributes critical tissue level information. Diffusion tensor imaging (DTI) reveals local collagen fiber architecture in the annulus fibrosus of intervertebral discs. This allows element-wise assignment of fiber directions that reflect actual tissue structure rather than idealized patterns [75]. Proton-density weighted sequences quantify tissue composition and hydration status, which directly influences material properties. This multimodal integration produces models where geometry, composition, and fiber architecture all derive from patient specific imaging [75,76]. This creates representations which was adapt as tissues degenerate or heal. A future direction is to use these modalities to create “living” FE models that update longitudinally. Traditional models represent a single time point but spinal biomechanics are constantly evolving because of continuous degeneration, healing, and adaptation. Serial imaging interval during recovery or disease progression can drive model updates which track changes in tissue properties, altered kinematics, and shifting distributions. For example, as fusion progresses, the increasing stiffness of fusion mass can be captured through follow-up CT scans and incorporated into updated models, while smart implant data validates predicted load transfer patterns [70].

The literature also points toward whole-spine and whole-body models becoming more common. Rather than isolating a small segment, researchers foresee using intact full-spine models to capture global effects (for example, how a lumbar fusion might subtly affect cervical spine mechanics due to posture changes) [14]. With increasing computational resources, such large-scale models may become feasible. These could integrate with gait analysis and muscle EMG data for a more complete picture of patient biomechanics pre- and post-surgery, guiding rehabilitation in addition to surgical choices. Ghezelbash et al., developed a subject-specific integrated FE-MS trunk model which combines detailed passive spinal structures with active muscular components. The model accurately predicted intradiscal pressure and muscle activities matching EMG trends across multiple subjects during functional movements [77]. This shift towards full-body modeling enables the prediction of compensatory mechanisms and reciprocal changes following spinal surgery. Full-body musculoskeletal models using inverse-inverse dynamics can predict postoperative sagittal alignment in response to spinal fusion with strong correlations between predicted and radiographic measures at follow-up [78]. Clinically, this could contribute to personalized surgical planning and outcome predication. Patient-specific models incorporating age- and pathology-related muscle deterioration, body hiatus, and treatment details can predict postoperative alignment and identify patients at high risk for sagittal imbalance or adjacent segment disease [79]. However, comprehensive validation remains essential. Recent systemic reviews emphasize that while certain modeling choices are standardized, considerable variability exists in muscular architecture representation and validation is often constrained by limited experimental data availability.

In summary, the coming years will likely see FEA becoming more personalized, through rapid patient-specific modeling and AI assistance; more comprehensive, by including more physiological factors and larger portions of the musculoskeletal system; and more accessible, via better interfaces and integration into clinical practice. These advancements, built on the solid foundation of current biomechanical understanding, promise to further bridge the gap between engineering simulations and patient care, ultimately improving surgical outcomes by leveraging the predictive power of FEA.

### 6.3. Limitations

This review should be interpreted within the context of several limitations. Included finite element studies demonstrated variability in model construction, material property assignment, boundary conditions, and loading paradigms, which limits direct quantitative comparison and instead supports trend-based synthesis. In addition, many models rely on simplified representations of physiologic loading and soft tissue behavior, which may not fully replicate in vivo spinal mechanics. Validation approaches were also inconsistently reported, with some studies incorporating experimental or clinical correlation and others relying on previously established modeling frameworks. Finally, as a narrative review, literature inclusion and synthesis were guided by thematic relevance rather than exhaustive systematic capture. This framework introduces the potential for selection bias and non-exhaustiveness, whereby relevant studies may not have been identified and included evidence may disproportionately reflect more commonly modeled constructs or more extensively reported surgical applications.

## 7. Conclusions

Finite element analysis has become an essential tool in spine surgery research, enabling evaluation of biomechanical consequences that are otherwise difficult to assess. This review highlights its role in surgical planning, including fusion approach selection, implant design, and prediction of postoperative challenges such as adjacent segment degeneration. The literature shows that FEA can compare interbody fusion techniques, quantify stress redistribution, and assess fixation devices and novel implants under physiological loads, directly informing strategies that optimize stability while minimizing implant failure and long-term degeneration risk. Finite element studies of anterior cervical constructs further demonstrate how plating systems, cage-screw combinations, and endplate preparation influence segmental stability, micromotion, and subsidence risk [80,81,82].

Model fidelity is critical. Patient-specific anatomy and realistic loading conditions produce more clinically relevant predictions, and validation against laboratory and clinical data remains necessary for reliability. Current limitations, including simplified material properties, exclusion of muscular forces, and technical expertise requirements, position FEA as a decision-support tool rather than a definitive predictor. However, ongoing computational advances are improving model speed, usability, and accuracy, moving toward routine patient-specific preoperative application.

For surgeons, engineers, and researchers, these developments are highly relevant. FEA informs operative planning, supports implant design innovation, and enables testing of biomechanical hypotheses such as the influence of sagittal alignment on adjacent level forces.

In conclusion, finite element modeling serves as a quantitative bridge between biomechanical theory and clinical reality. When validated and appropriately interpreted, it can enhance surgical planning and support patient-specific treatment strategies. As modeling and validation continue to evolve, FEA is positioned to transition from a research tool to an everyday clinical asset, with multicenter outcome validation representing a key next step.

## Figures and Tables

**Table 1:** Summary of finite element investigations included in this review, including modeled spinal region, surgical construct evaluated, model framework, loading paradigms, primary biomechanical outputs, and validation approaches.

Study	Spine Region	Surgical Procedure/Construct	Model Type	Loading Conditions	Primary Outputs	Validation
Shimooki et al., 2024 [1]	Lumbar (multilevel)	TLIF vs. PLIF vs. LLIF vs. PLF with posterior instrumentation	CT-based lumbar fusion FE model	Physiologic spinal loading across motion states	Rod stress; pedicle screw stress	Not specified
Oikawa et al., 2022 [2]	Lumbar	LLIF vs. PLIF vs. TLIF implant failure comparison	Lumbar fusion FE model	Multiplanar physiologic loading	Implant stress; failure susceptibility	Not specified
Xu et al., 2024 [3]	Cervical	Skip-level ACDF strategies	Cervical FE model	Flexion, extension, rotation	Adjacent ROM; disc pressure	Not specified
Stoner et al., 2020 [16]	Cervical	ACDF vs. laminoplasty	Surgical cervical FE model	Physiologic cervical loading	Cord strain; segment motion	Not specified
Sensale et al., 2021 [5]	Lumbar vertebrae	Pedicle screw size and geometry	Patient-specific vertebral FE model	Pullout loading	Screw stress; bone strain	Experimental correlation
Nikkhoo et al., 2020 [13]	Lumbar	Lordosis angle effects in fusion surgery	Parametric patient-specific FE model	Physiologic lumbar loading	Adjacent segment stress; fusion mechanics	Not specified
Nevzati et al., 2024 [15]	Thoracolumbar	Lateral corpectomy vs. posterior instrumentation	Thoracolumbar FE construct model	Physiologic loading	Construct stability; stress distribution	Not specified
Son et al., 2022 [17]	Lumbar deformity	Multilevel instrumented fusion level selection	Lumbar deformity FE model	Physiologic motion loading	Junctional stress; construct biomechanics	Not specified
Lu et al., 2019 [18]	Lumbar	PLF vs. TLIF vs. OLIF vs. XLIF	Lumbar interbody FE model	Physiologic loading	Segmental ROM; implant stress	Not specified
Wang et al., 2023 [19]	Lumbar	Dynamic vs. rigid fixation	Lumbar fixation FE model	Sagittal alignment loading	Disc stress; implant load; ROM	Not specified
Azadi & Arjmand, 2021 [20]	Lumbar	Post-fusion adjacent segment effects	Validated lumbar FE model	Physiologic loading	Adjacent disc stress; ROM	Experimental validation framework
Wang et al., 2024 [21]	Thoracolumbar deformity	Adult scoliosis correction fusion	Deformity correction FE model	Physiologic loading	Adjacent segment biomechanics	Not specified
van Rijsbergen et al., 2018 [22]	Lumbar adjacent levels	Post-fusion degeneration prediction	Patient-specific mechanobiologic FE model	Time-dependent simulation	Disc degeneration; bone density	Clinical correlation
Nguyen et al., 2024 [23]	Thoracolumbar	T10–pelvis fusion complications	Population-based subject-specific FE models	Standing physiologic loading	Junctional shear; compressive force	Not specified
Mischler et al., 2026 [24]	Lumbar vertebra	CF/PEEK pedicle screw fixation	Micro-FE + experimental model	Pullout loading	Screw pullout strength	Experimental validation
Hsieh et al., 2022 [25]	Lumbar	Hybrid elastic rod fracture	Lumbar fixation FE model	Physiologic loading	Rod stress; fracture mechanics	Not specified
Ye et al., 2023 [26]	Lumbar	Pedicle screw reposition strength	Vertebral FE model	Pullout loading	Fixation strength	Not specified
Kiapour et al., 2022 [27]	Lumbar interbody	Truss-based fusion device	Implant FE + in vivo model	Axial loading	Cage strain; fusion response	Animal validation
Lu et al., 2022 [28]	Lumbar TLIF	Cage modulus variation	Parametric FE simulation	Physiologic loading	Endplate stress; rod stress	Regression modeling
Groenen et al., 2018 [29]	Lumbar functional units	Vertebral failure prediction	Nonlinear FE model	Failure loading	Fracture risk; load tolerance	Cadaver comparison
George et al., 2023 [30]	Lumbar	Customizable fusion modeling	Patient-specific lumbar FE model	Physiologic loading	Construct biomechanics	Model validation
Yu et al., 2025 [31]	Lumbar	Patient-specific cage design	Morphable lumbar FE pipeline	Physiologic loading	Cage stress; segment biomechanics	Not specified
Shash et al., 2025 [32]	Lumbar (L4–L5)	Interspinous vs. interbody devices	AI-integrated FE model	Physiologic loading	Implant stress; motion preservation	Not specified
Ahmadi et al., 2025 [33]	Lumbar	Automated spine modeling	Automated segmentation FE workflow	Physiologic loading	Load distribution; stress mapping	Workflow validation

**Table 2:** Advantages and Limitations of FEA in Clinical Practice.

Aspect	Advantage	Limitation
Virtual Testing	Simulates surgical options without patient risk (ex. can test multiple fusion strategies preoperatively).	Requires significant expertise/software to set up models. Time-consuming (days per simulation).
Quantitave Testing	Measures internal stresses/strains not observable in vivo (ex. identifies stress points explaining hardware failure.	Often uses simplifying assumptions (linear material, no muscle), which may reduce accuracy in complex cases.
Patient Specificity	Can tailor models to individual anatomy and pathology (ex. CT-based models reflect a patient’s bone quality and alignment).	Historically relied on generic models. Personalized modeling is emerging but not yet routine in clinics.
Implant Design Insight	Pre-tests new devices under load (ex. optimized lattice cage design before clinical use).	May not capture all physiological factors (healing response, patient variability). Validated mostly on mechanical outcomes, less on long-term biological effects.
Overall Utility	Provides a “dry run” of surgery—predictive, visual, and risk-free tool to refine plans.	Key Challenge: Integration into workflow—needs to be faster, easier, and proven reliable to gain widespread adoption.

## Data Availability

No new data were created or analyzed in this study.

## Abbreviations

The following abbreviations are used in this manuscript:

ACDF	Anterior cervical discectomy and fusion
AI	Artificial intelligence
AI-FEA	Artificial intelligence–enhanced finite element analysis
ASD	Adjacent segment degeneration
FEA	Finite element analysis
LLIF	Lateral lumbar interbody fusion
PEEK	Polyetheretherketone
PJK	Proximal junctional kyphosis
PLF	Posterolateral fusion
PLIF	Posterior lumbar interbody fusion
ROM	Range of motion
TLIF	Transforaminal lumbar interbody fusion
